# Percutaneous endoscopic decompression for lumbar spinal stenosis: Protocol for a systematic review and network meta-analysis

**DOI:** 10.1097/MD.0000000000015635

**Published:** 2019-05-17

**Authors:** Jianjun Liu, Hongwei Zhang, Xiaogang Zhang, Tao He, Xiyun Zhao, Zhipeng Wang

**Affiliations:** a Department of Orthopaedic, Affiliated Hospital of Gansu University of Traditional Chinese Medicine; b Clinical College of Chinese Medicine, Guansu University of Traditional Chinese Medicine; c Gansu Provincial Rehabilitation Central Hospital, Lanzhou, China.

**Keywords:** lumbar spinal stenosis, network meta-analysis, percutaneous endoscopic decompression, systematic review

## Abstract

**Background:**

Lumbar spinal stenosis (LSS) is a common and frequently-occurring disease in clinical practice. There are many interventions to treat it, and percutaneous endoscopic decompression (PED) is one of them, but their relative efficacy and safety remains unclear. Hence, the present study aims to synthesize the available direct and indirect evidence on the PED and other treatments for LSS.

**Methods:**

The following databases will be searched: Cochrane Library, PubMed, Web of Science, Embase and China Biomedical Literature Database (CBM). The search dates will be set from the inception to April 2019. All randomized controlled trials (RCTs) will be included in this network meta-analysis (NMA) and their risk of bias will be assessed using Cochrane handbook tool by 2 independent authors. The efficacy outcomes including: Back and Leg Visual Analog Scale (VAS) score, MacNab criteria, the Oswestry Disability Index (ODI), and Japanese Orthopedic Association (JOA) score. The safety outcomes including: incidence of complications (dura tear, incomplete decompression, reoperation, etc.). A network meta-analysis will be performed using R x64 3.5.1 software and pairwise meta-analysis will be conducted using Stata 12.0 software. Grading of recommendations assessment, development, and evaluation (GRADE) will be used to assess evidence quality.

**Results:**

The results of NMA will be submitted to a peer-reviewed journal.

**Conclusion:**

The NMA will provide a comprehensive evidence summary on treatments for patients with LSS.

**Protocol registration number:**

CRD42019120509.

## 1 Introduction

Lumbar spinal stenosis (LSS) is a common degenerative disease of the spine, which is one of the common causes of functional disorders such as lumbar leg pain and neurogenic intermittent claudication.^[1]^ About one-fifth of patients over 65 years old have symptoms of neurogenic intermittent claudication, and this disease has become the most common cause of spinal surgery in patients over 65 years old, which significantly affects the activity ability and quality of life of patients.^[2]^ At present, there are many surgical or non-surgical treatments for LSS.^[3–6]^ Especially, with the development of the techniques of percutaneous endoscopic lumbar spine surgery, percutaneous endoscopic decompression (PED) has gradually developed into an alternative for the treatment of LSS.^[7]^ But, their relative efficacy and safety are unclear, and to the best of our knowledge, there is not a study to compare their relative efficacy and safety, then there is a big obstacle for clinicians to select them reasonably.

Network meta-analysis (NMA) is an effective method to compare ≥3 interventions for a specific disease.^[8]^ And recent years, it has been adopted widely by researchers and clinicians, and using it to assess the relative effectiveness by compare multiple interventions simultaneously.^[9]^ And eventually, it will generate a ranking resultby using all of available interventions, and the result will be used to guide the clinical practice.^[10]^

Therefore, we designed a NMA to compare the efficacy and safety of all available treatments for LSS, and we hope the results from our present study will provide reference to clinical practice.

## 2 Methods

### 2.1 Study registration

This study protocol has been registered on PROSPERO: CRD42019120509.

### 2.2 Eligibility criteria

#### 2.2.1 Type of study

Randomized controlled trials (RCTs) that compared the effect of PED for LSS with other treatments will be included in this NMA. And no any limitation will be used for the study. We will exclude publications that were not peer-reviewed or cannot retrieve relevant data, such as letters, comments, and conference proceedings.

#### 2.2.2 Participants

We will include patients with lumbar spinal stenosis were diagnosed using any recognized diagnostic criteria, such as the evidence-based clinical guideline on the diagnosis and treatment of degenerative lumbar spinal stenosis by the North American Spine Society (NASS).^[11]^ But, patients with a lumbar surgery history, infection, tuberculosis, tumors, and other diseases will be excluded.

#### 2.2.3 Interventions

Percutaneous endoscopic decompression (PED), methodologically defined as follows: a thin working sheath is completely inserted percutaneously through a stab incision. A working-channel endoscope is then placed in the working sheath.^[12]^ Surgical instruments are then introduced through the working channel. The surgical field is always visualized using a monitor system. The procedure is performed under continuous saline irrigation.

PED including interlaminar PED via the posterior interlaminar approach is mainly used for the decompression of central and/or lateral recess stenosis. Transforaminal PED via the lateral approach is suitable for lateral recess stenosis with or without foraminal stenosis. Finally, endoscopic lumbar foraminotomy via the posterolateral extraforaminal approach is adequate for foraminal or extraforaminal stenosis. Percutaneous endoscopic decompression combined with lumbar interbody fusion will be excluded.

We will include studies that compared PED to other treatments for LSS, others treatments including common analgesics, spinal manipulative therapy, or other surgical interventions.

### 2.3 Outcomes

The efficacy outcomes including: Back and Leg Visual Analog Scale (VAS) score,^[13]^ MacNab criteria,^[14]^ the Oswestry Disability Index (ODI),^[15]^ and Japanese Orthopedic Association (JOA) score.^[16]^ The safety outcomes including: incidence of complications (dura tear, incomplete decompression, reoperation, incidental durotomy, epidural hematoma, headache, infection, recurrence rate). RCTs reporting above at least 1 outcome will be included the present study.

### 2.4 Data source

The following databases will be searched: The Cochrane Library, PubMed, Web of Science, Embase, and China Biomedical Literature Database (CBM). In addition, we will also examine the reference lists of all eligible articles for potential available studies. The search dates will be set from the inception to April 2019. The searching strategy of PubMed is as follows:

#1 “Constriction, Pathologic”[Mesh]

#2 “Lumbar Vertebrae”[Mesh]

#3 “Spinal Canal”[Mesh]

#4 “Spinal Diseases”[Mesh]

#5 “Spinal Stenosis”[Mesh]

#6 “Spinal Osteophytosis”[Mesh]

#7 “Spondylosis”[Mesh]

#8 “Cauda Equina”[Mesh]

#9 lumb∗ [Title/Abstract]

#10 spin∗[Title/Abstract]

#11 stenosis [Title/Abstract]

#12 spondyl∗[Title/Abstract]

#13 osteophytosis [Title/Abstract]

#14 “neurogenic claudication”[Title/Abstract]

#15 “lumbar radicular pain” [Title/Abstract]

#16 #11 OR #12

#17 #9 AND #16

#18 #11 OR #13

#19 #10 AND #18

#20 #1 OR #2 OR #3 OR #4 OR #5 OR #6 OR #7 OR #8 OR #14 OR #15 OR #17 OR #19

#21 “Randomized Controlled Trial” [Publication Type]

#22 “Controlled Clinical Trial” [Publication Type]

#23 “Pragmatic Clinical Trial” [Publication Type]

#24 “Clinical Trial” [Publication Type]

#25 “Placebos”[Mesh]

#26 random∗[Title/Abstract]

#27 trial∗[Title/Abstract]

#28 blind [Title/Abstract]

#29 placebo [Title/Abstract]

#30 #21 OR #22 OR #23 OR #24 OR #25 OR #26 OR #27 OR #28 OR #29

#31 #20 AND #30

### 2.5 Study selection

**Figure 1:**
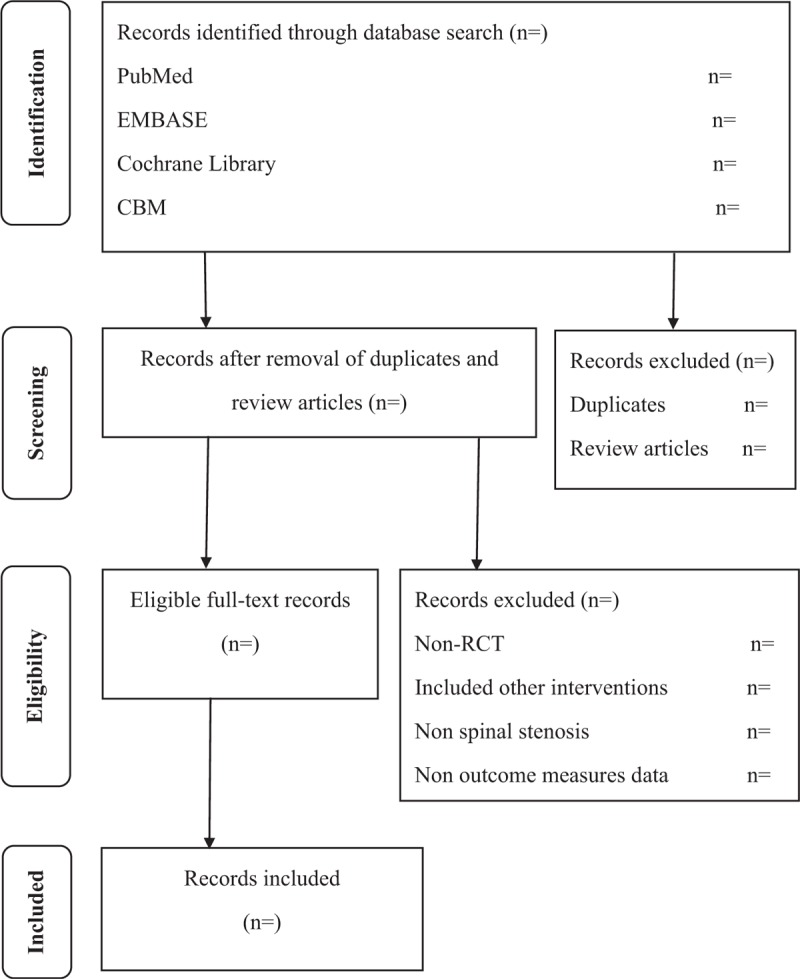
Process of study search and selection.

The Endnote X7 will be used to manage hits from all of databases. Screening process will include 2 stages, first, 2 experienced reviewers will independently check the title and abstract of all hits and to find appropriate studies according to our eligibility criteria; second, each full text from first stage will be downloaded and to further check (Fig. 1). Any disagreement will be resolved through discussion. A detailed data-extracted form will be established using Microsoft Excel 2016 (Microsoft, WA), and mainly information including: first author, year of study, sample size, patient characteristics, interventions, therapeutic regimens, and outcomes. The third author will examine all extracted information to decrease bias. For continuous outcomes, when the data were reported as median rather than mean, and range or interquartile rather than standard deviation, the mean and standard deviation will be estimated using method from Hozo et al.^[17]^

### 2.6 Risk of bias (ROB) assessment

Two reviewers will independently assess the ROB for all included studies using the Cochrane handbook tool.^[18]^ And this tool consists of 6 domains: random sequence generation, allocation concealment, blind, incomplete outcome data, selective reporting, and other bias. The process also will be implemented by 2 reviewers independently and any difference through discussion to reach agreement.

### 2.7 Statistical analysis

#### 2.7.1 Pairwise meta-analyses

The Stata 12.0 software (StataCorp, College Station, TX) will be used to perform pairwise meta-analyses with random-effects model. Dichotomous outcomes will be measured using relative risk (RR) with 95% confidence interval (95% CI), and the mean difference (MD) with 95% CI will be used to present continuous outcomes. The potential heterogeneity will be measured using *I*^2^, when the *I*^2^ >50% and *P *< .1, subgroup analysis will be performed to explore the heterogeneity. Publication bias will be tested using Begg and Egger funnel plot when the number of included studies for 1 special outcome are not <10.^[19]^

#### 2.7.2 Network meta-analyses

The R x64 3.5.1 software will be used to performed a Bayesian NMA. The inconsistency between direct and indirect comparisons will be tested using node splitting method.^[20]^ Surface under the cumulative ranking area (SUCRA) will be used to rank the different interventions for patients with LSS. Network geometry will use nodes to represent different treatments and edges to represent the head-to-head treatments. And the size of node represents sample sizes of intervention, thickness of edge represents numbers of included studies.

### 2.8 Quality of evidence

The quality of evidence for all outcomes will be assessed using the grading of recommendations assessment, development, and evaluation (GRADE)^[21]^ mainly considerations including: risk of bias, inaccuracy, inconsistency, indirectness, publication bias, and results of assessment will be graded 4 levels: very low, low, moderate, and high level.

## 3 Discussion

To the best of our knowledge, this is the first network meta-analysis to compare PED to other available interventions for LSS. So, the present NMA will fill this gap according to Cochrane Handbook and the preferred reporting items for systematic review and meta-analysis (PRISMA) extension statement for NMAs. The study will provide an available direct and indirect evidence on the PED and other treatments for LSS, and to generate a treatment ranking based on their efficacy and safety outcomes. The present protocol was designed in adherence to PRISMA-P^[22,23]^ which is used to report a protocol of systematic review with or without meta-analysis.

## Author contributions

**Data curation:** Jianjun Liu, Hongwei Zhang, Zhipeng Wang.

**Formal analysis:** Hongwei Zhang.

**Funding acquisition:** Xiaogang Zhang.

**Methodology:** Xiaogang Zhang, Tao He, Xiyun Zhao, Zhipeng Wang.

**Resources:** Jianjun Liu, Xiaogang Zhang, Xiyun Zhao.

**Software:** Tao He, Xiyun Zhao, Zhipeng Wang.

**Writing – original draft:** Jianjun Liu, Zhipeng Wang.

**Writing – review & editing:** Jianjun Liu, Hongwei Zhang.
